# Multicollinear physical activity accelerometry data and associations to cardiometabolic health: challenges, pitfalls, and potential solutions

**DOI:** 10.1186/s12966-019-0836-z

**Published:** 2019-08-27

**Authors:** Eivind Aadland, Olav Martin Kvalheim, Sigmund Alfred Anderssen, Geir Kåre Resaland, Lars Bo Andersen

**Affiliations:** 1 Faculty of Education, Arts and Sports, Department of Sport, Food and Natural Sciences, Campus Sogndal, Western Norway University of Applied Sciences, Box 133, 6851 Sogndal, Norway; 2 Department of Chemistry, University of Bergen, Box 7800, 5020 Bergen, Norway; 3 Department of Sports Medicine, Norwegian School of Sport Sciences, Box 4014, Ullevål Stadion, 0806 Oslo, Norway; 4 Faculty of Education, Arts and Sports, Department of Sport, Center for Physically Active Learning, Campus Sogndal, Western Norway University of Applied Sciences, Box 133, 6851 Sogndal, Norway

**Keywords:** Multivariate pattern analysis, Compositional data analysis, Multiple linear regression, Multicollinearity, Statistics, Children, Accelerometer, Intensity

## Abstract

**Background:**

The analysis of associations between accelerometer-derived physical activity (PA) intensities and cardiometabolic health is a major challenge due to multicollinearity between the explanatory variables. This challenge has facilitated the application of different analytic approaches within the field. The aim of the present study was to compare association patterns of PA intensities with cardiometabolic health in children obtained from multiple linear regression, compositional data analysis, and multivariate pattern analysis.

**Methods:**

A sample of 841 children (age 10.2 ± 0.3 years; BMI 18.0 ± 3.0; 50% boys) provided valid accelerometry and cardiometabolic health data. Accelerometry (ActiGraph GT3X+) data were characterized into traditional (four PA intensity variables) and more detailed categories (23 PA intensity variables covering the intensity spectrum; 0–99 to ≥10,000 counts per minute). Several indices of cardiometabolic health were used to create a composite cardiometabolic health score. Multiple linear regression and multivariate pattern analyses were used to analyze both raw and compositional data.

**Results:**

Besides a consistent negative (favorable) association between vigorous PA and the cardiometabolic health measure using the traditional description of PA data, associations between PA intensities and cardiometabolic health differed substantially depending on the analytic approaches used. Multiple linear regression lead to instable and spurious associations, while compositional data analysis showed distorted association patterns. Multivariate pattern analysis appeared to handle the raw PA data correctly, leading to more plausible interpretations of the associations between PA intensities and cardiometabolic health.

**Conclusions:**

Future studies should consider multivariate pattern analysis without any transformation of PA data when examining relationships between PA intensity patterns and health outcomes.

**Trial registration:**

The study was registered in Clinicaltrials.gov 7th of April 2014 with identification number NCT02132494.

**Electronic supplementary material:**

The online version of this article (10.1186/s12966-019-0836-z) contains supplementary material, which is available to authorized users.

## Background

Accelerometer-derived physical activity (PA) is often broadly represented across a spectrum of time spent in different intensities (sedentary (SED), light PA (LPA), moderate PA (MPA), vigorous PA (VPA) and/or moderate-to-vigorous PA (MVPA). However, most studies investigating associations between PA and cardiometabolic health have targeted only selected parts of this spectrum. In children, there is strong evidence for an association between time spent in MVPA and VPA and cardiometabolic health outcomes, and weaker associations for lower intensity PA [1–4]. However, few studies incorporate the entire intensity spectrum. This is important as focusing only on selected parts of it leads to a loss of information from accelerometry data and it creates at least two problems for interpretation of study results: 1) It ignores the possible influence of other intensities on health and 2) it increases susceptibility of residual confounding [4–6]. Accordingly, associations across the whole PA intensity spectrum should be examined to obtain a complete picture and to facilitate improved interpretations of how PA relates to health outcomes [4–7].

Strong multicollinearity between intensity variables across the PA spectrum, represents a major limitation for common statistical methods such as ordinary least squares multiple linear regression [8]. Thus, statistical approaches that can overcome this challenge are needed [7, 9]. A number of different analytic approaches are now being incorporated in the field, including isotemporal substitution models [10, 11], compositional data analysis [12, 13], and multivariate pattern analysis [6, 14]. In the following section, we provide a brief overview of the analytical challenges of modelling associations for the PA intensity spectrum (obtained from accelerometry) as explanatory variables with a given outcome, and how different statistical approaches are applied to address these challenges.

### Current approaches used to address multicollinearity in physical activity data

The multicollinearity challenge encountered when analyzing the full PA intensity spectrum has two aspects: First, the measured PA behaviors (i.e., time spent in different intensities) are inherently related; SED is negatively correlated with PA (non-SED activity), whereas other PA intensities are positively related to each other [6]. Second, the derived variables have a closed structure, caused by the fact that behaviors substitute each other within a finite period of time. If including sleep, variables sum to 24 h; otherwise, variables sum to individuals’ total accelerometer wear time (i.e., 100%). Because the total time budget is fixed, all behaviors increase (or decrease) at the expense of others, which means time is always reallocated among variables [7]. To account for this property of the data, one could adjust for total wear time in the statistical model or analyze proportions of the total sum (e.g., by normalization to 24 h or to total wear time). However, both solutions cause singularity of the explanatory variables induced through “closure” of the dataset (i.e., the total correlation within the data matrix is − 1), which leads to data violating the assumptions of multiple linear regression. Imagine having a simple dataset with two variables, for example SED and non-SED PA: Given a constant sum of these variables, they will be perfectly negatively correlated and thus singular.

Isotemporal substitution models (a special case of multiple linear regression) was introduced in the field of PA epidemiology by Mekary et al. [10] in 2009 to solve the “closure” or “constant-sum” challenge by removing one of the explanatory variables at-a-time from the model. For instance, if modelling the associations of the four explanatory variables SED, LPA, MPA, and VPA, four different models are developed, each one including only three of the PA variables together with total wear time. Associations between the remaining explanatory variables and the outcome are then interpreted as the theoretical effect of reallocating time from the excluded variable to the included variables (e.g., from SED to MVPA). There are several challenges with this model. Although it addresses the singularity problem posed by closure, the remaining variables are still multicollinear, which violates the assumptions of linear regression. Moreover, the model may be complex to interpret, because of the multiple iterations. Resultant reallocations are also rather theoretical, since a change in any given variable in practice would still be related to all the other PA intensities, not only one. Importantly, these challenges increase substantially when applied to a dataset with greater resolution. That is, when attempting to incorporate data across the entire PA intensity spectrum [6] as opposed to more blunt descriptions (i.e., SED, LPA, MVPA). Due to its similarity to multiple linear regression models and additional limitations, we do not address isotemporal substitution models in this paper.

In order to acknowledge that PA data has a closed structure, Pedisic et al. [7] and Chastin et al. [12] introduced compositional data analysis into PA research in 2014–2015. In contrast to isotemporal substitution models, where reallocation of time is related to one variable at a time, compositional data analysis first transforms all variables into compositions and log-transforms them. Each variable is then given as a ratio to the geometric mean of other variables. Compositional data analysis, or log-ratio methods, were developed to solve the closure problem for geochemical applications where the variables summed to a constant, for example 100% [15]. The standard approach, the centered log-ratio (clr) method, was proposed by Aitchison in 1982 [15]. The clr method transforms the normalized variables in a symmetric way by expressing them as their log-ratios to the geometric mean of all the explanatory variables. However, the clr method does not solve the problem of singularity in the dataset, and thus the data violates the assumptions of multiple linear regression. To avoid this problem and be able to analyze data using linear regression, several different isometric log-ratio (ilr) methods have been developed, which through a set of transformations, in contrast to clr, in principle provides an open dataset [12, 16]. Compositional data analysis methods are increasingly being applied in PA and SED behavior research. However, interpretation of regression coefficients from these models remains a key challenge and several other important limitations exist. Early studies have shown that the underlying correlation structure of the dataset can be significantly distorted by normalization, depending on the means, variances and number of the explanatory variables [17, 18]. Specifically, great variation in the means and variances among variables, and few variables included in the model, can result in great distortion. Log-transformation and centering can further exaggerate this distortion induced by normalization [19]. Thus, distortion of the correlation structure among the PA variables is likely when compositional analysis is applied to a traditional PA dataset where means and variances differ greatly among only a selection of variables (SED, LPA, MPA, VPA, MVPA and/or sleep). This distortion is likely to influence the association patterns with a given outcome. As such, the application of compositional data analysis models requires further consideration.

Aadland et al. [6, 14] recently addressed the multicollinearity challenge of accelerometer-derived PA data using multivariate pattern analysis. This method is widely applied in pharmaceutical [20] and metabolomics studies [21], in addition to other fields of biomedical research, such as in treatment and diagnosis of diseases [22], with the objective of revealing patterns of important biomarkers among hundreds or thousands of highly interrelated variables. As previously called for [4, 5], this statistical method can handle completely collinear explanatory variables by combining the data into orthogonal latent variables (also see further details in the Methods) [23]. In this way, it treats accelerometry-derived PA variables as an intensity spectrum – described by any number of exploratory variables – without requiring any data transformation. Aadland et al. [6] applied 16 PA intensity intervals between 0 and 99 and ≥ 8000 counts per minute (cpm), and found that intensities in the vigorous range (5000–7000 cpm) were strongest associated with cardiometabolic health, while MPA (approximately 2000 to 4000 cpm) was weakly associated with health, and SED (< 100 cpm) and LPA (100 to approximately 2000 cpm) were not associated with health. Thus, multivariate pattern analysis can provide a more detailed interrogation of PA data across the entire intensity spectrum while also greatly improving knowledge of multivariate association patterns – the signature – of PA related to cardiometabolic health [6].

### Aims

Results from the different analytic approaches discussed above have not been directly compared. Therefore, the aim of this analysis was to compare the use of multiple linear regression and multivariate pattern analysis methods, as applied to raw (untransformed) data and compositional (log-ratio transformed) data, with regard to associations between PA and cardiometabolic health in children. All models were applied to the same underlying dataset, but with different descriptions of PA intensities. Specifically, one description used four” traditional” PA intensities (SED, LPA, MPA, and VPA) and the other description used greater resolution, including 23 PA intensity variables covering the whole intensity spectrum (0–99 to ≥10,000 cpm).

## Methods

### Participants

We used baseline data obtained from fifth-grade children in the Active Smarter Kids (ASK) cluster-randomized controlled trial, conducted in the County of Sogn og Fjordane, Norway during 2014–2015 [24, 25]. Sixty schools, encompassing 1202 fifth-grade children, fulfilled the inclusion criteria, and agreed to participate. This sample represented 86.2% of the population of 10-year-olds in the county, and 95.2% of those eligible for recruitment. Later, three schools declined to participate. Thus, 1145 (97.4%) of 1175 available children from 57 schools agreed to participate in the study.

Our procedures and methods conform to ethical guidelines defined by the World Medical Association’s Declaration of Helsinki and its subsequent revisions. The South-East Regional Committee for Medical Research Ethics in Norway approved the study protocol. We obtained written informed consent from each child’s parents or legal guardian and from the responsible school authorities prior to all testing. The study is registered in Clinicaltrials.gov with identification number: NCT02132494.

### Procedures

We have previously published a detailed description of the study [24], and therefore provide only a brief overview of the relevant procedures herein.

#### Physical activity

PA was measured using the ActiGraph GT3X+ accelerometer (Pensacola, FL, USA) [26]. Participants were instructed to wear the accelerometer at the waist at all times over seven consecutive days, except during water activities (swimming, showering) or while sleeping. Units were initialized at a sampling rate of 30 Hz. Files were analyzed at 1-s epochs to capture low and high intensity PA [14, 27] using the KineSoft analytical software version 3.3.80 (KineSoft, Loughborough, UK). Data were restricted to hours 06:00 to 23:59. In all analyses, consecutive periods of ≥ 60 min of zero counts were defined as non-wear time [28]. We applied wear time requirements of ≥ 8 h/day and ≥ 4 days/week to constitute a valid measurement [29].

We compared the different statistical approaches using two different descriptions of the PA data; a “traditional” description consisting of four PA intensity variables and a “spectrum” description including 23 PA intensity variables across the intensity spectrum. We created the first description using the Evenson et al. [30, 31] cut points of 0–99, 100–2295, 2296–4011, ≥ 4012 cpm to determine SED, LPA, MPA, and VPA. Additionally, MVPA (≥ 2296 cpm) and the proportion of children achieving the guideline PA level (mean of ≥ 60 min MVPA/day) was reported for descriptive purposes. We created the latter description using 23 PA variables of total time (min/day) to capture movement in narrow intensity intervals across the activity spectrum; 0–99, 100–249, 250–499, 500–999, 1000–1499, 1500–1999, 2000–2499, 2500–2999, 3000–3499, 3500–3999, 4000–4499, 4500–4999, 5000–5499, 5500–5999, 6000–6499, 6500–6999, 7000–7499, 7500–7999, 8000–8499, 8500–8999, 9000–9499, 9500–9999, and ≥ 10,000 cpm. This approach is similar to the approach used by Aadland et al. [6], but extends the intensity spectrum in the vigorous intensity range.

#### Cardiometabolic health outcome measures

Aerobic fitness was measured with the Andersen intermittent running test, which has demonstrated acceptable reliability and validity in 10-year-old children [32]. Children ran as long as possible in a to-and-fro movement on a 20-m track, touching the floor with a hand each time they turned, with 15-s work periods and 15-s breaks, for a total duration of 10 min. The distance (meters) covered was used as the outcome. Body mass was measured to the nearest 0.1 kg using an electronic scale (Seca 899, SECA GmbH, Hamburg, Germany) with children wearing light clothing. Height was measured to the nearest 0.1 cm using a portable Seca 217 (SECA GmbH, Hamburg, Germany). Body mass index (BMI) (kg ·m^− 2^) was calculated. Waist circumference was measured to the nearest 0.1 cm with a Seca 201 (SECA GmbH, Hamburg, Germany) ergonomic circumference measuring tape two cm over the level of the umbilicus. Systolic (SBP) and diastolic blood pressures were measured using the Omron HBP-1300 automated blood pressure monitor (Omron Healthcare, Inc., Vernon Hills, IL, US). Children rested quietly for ten minutes in a sitting position with no distractions before blood pressures was measured four times; we used the mean of the last three measurements for analyses. Serum blood samples were collected from the children’s antecubital vein between 08:00 and 10:00 in the morning after an overnight fast. All blood samples were analyzed for total cholesterol (TC), triglyceride (TG), high-density lipoprotein cholesterol (HDL), glucose, and insulin at the accredited Endocrine Laboratory of the VU Medical Center (VUmc; Amsterdam, the Netherlands). Low-density lipoprotein cholesterol (LDL) was estimated using the Friedewald formula [33]. We calculated the TC:HDL ratio and homeostasis model assessment (HOMA) (glucose (mmol/L) * insulin (pmol/L) / 22.5) [34].

We calculated a composite cardiometabolic health score as the mean of six variables (SBP, TG, TC:HDL ratio, HOMA, waist:height ratio, and the reversed Andersen test) by averaging standardized scores after adjustment for sex and age. A higher score indicates higher risk. A similar approach have been used previously [2, 6]. This composite score was used as the outcome in all models.

### Statistical analyses

Children’s characteristics were reported as frequencies, means, and standard deviations (SD). We tested for differences in characteristics between boys and girls, as well as between included and excluded children, using a linear mixed model to account for the clustering among studies. Models for PA and SED were adjusted for wear time.

We used Pearson’s correlation coefficients (r) to analyze the correlation structure among the explanatory variables (PA) and to analyze bivariate associations between PA and cardiometabolic health. Thereafter, associations between PA and cardiometabolic health were determined using four different models: 1) multiple linear regression of raw (untransformed) data, 2) multiple linear regression of compositional (ilr-transformed) data, 3) multivariate pattern analysis of raw data, and 4) multivariate pattern analysis of compositional (clr-transformed) data.

#### Compositional transformation of data

Compositional transformation of PA data was performed using the clr and the ilr methods as described by Hron et al. [16]. In both transformations, variables were normalized (all explanatory variables summing to 1) prior to making natural log-transformation of each variable. Using the clr method, each transformed variable was centered according to the mean logarithm of all explanatory variables [15]. As this approach implies singularity of the dataset (i.e., it induces a correlation of − 1 spread over the explanatory variables), which makes it unsuitable for analysis using linear regression, these data were analyzed using multivariate pattern analysis, which can handle singular data. Using the ilr-transformation, each transformed variable was centered to the mean logarithm of all the following explanatory variables after successively removing the variables being transformed one at a time. The procedure was repeated after permuting such that all explanatory variables have been the first variable once [16]. As this approach technically provides an open dataset (i.e., it does not impose the spurious correlation of − 1 and thus singularity), these datasets were analyzed using multiple linear regression, repeated as many times as the number of explanatory variables (i.e., four and 23; once for each permutation of the explanatory variables). The procedure of repeating the analysis after permutation when using ilr-transformed data is neither necessary nor suitable for multivariate pattern analysis because this model can handle singular data and because the correct multivariate association pattern cannot be determined from one joint interpretable model.

#### Multiple linear regression

We included all PA variables as explanatory variables and the composite cardiometabolic health score as the outcome variable. We reported regression coefficients and their 95% confidence intervals (CI). These analyses were performed using IBM SPSS v. 24 (IBM SPSS Statistics for Windows, Armonk, NY: IBM Corp., USA).

#### Multivariate pattern analysis

Partial least squares (PLS) regression analysis [23] was used to determine the multivariate association pattern of PA with cardiometabolic health. We included all PA variables as explanatory variables and the composite cardiometabolic health score as the outcome variable, as shown previously [6]. PLS regression decomposes the explanatory variables into orthogonal linear combinations (PLS components), while simultaneously maximizing the covariance with the outcome variable. Thus, PLS regression is able to handle completely collinear variables through the use of latent variable modelling [23]. The procedure differs from that of factor analysis or principal component analysis by creating components that maximize the covariation with the outcome, not internally among the explanatory variables. Prior to PLS regression, all variables were centered and standardized to unit variance. Models were cross-validated using Monte Carlo resampling [35] with 1000 repetitions by repeatedly and randomly keeping 50% of the subjects as an external validation set when estimating the models. For each validated PLS regression model, a single predictive component was subsequently calculated by means of target projection [20, 36] to express all the predictive variance in the PA intensity spectrum related to cardiometabolic health in a single intensity vector. Selectivity ratios (SRs) with 95% CIs were obtained as the ratio of this explained predictive variance to the total variance for each PA intensity variable [37, 38]. The procedure for obtaining the multivariate patterns is completely data-driven, with no assumptions on variable distributions or degree of collinearity among variables. These analyses were performed by means of the commercial software Sirius version 11.0 (Pattern Recognition Systems AS, Bergen, Norway).

## Results

### Children’s characteristics

We included 841 children (50% boys) who provided valid data on all relevant variables (Table 1). The children included in the present analyses did not differ from the excluded children (*n* = 288, 57% boys) with respect to age (*p* ≥ .689) or anthropometry (*p* ≥ .166). However, the included children performed better on the Andersen test (mean 898 (95% CI; 891–905) vs. 870 (856–884) meter, *p* < .001), had lower fasting insulin concentrations (55.0 (52.9–57.0) vs. 64.5 (57.1–71.8) pmol/l, *p* = .001) and HOMA scores (1.71 (1.64–1.78) vs. 2.02 (1.78–2.27), *p* = .002), exhibited less SED time (597 (593–601) vs. 607 (598–615) min/day, *p* = .002), and spent more time in in LPA (122 (121–124) vs. 118 (115–121) min/day, *p* = .015), MPA (37 (36–38) vs. 35 (34–37) min/day, *p* = .010), VPA (39 (38–40) vs. 36 (33–38) min/day, *p* = .005), and MVPA (76 (75–78) vs. 71 (67–74) min/day, *p* = .003) than the excluded children.

**Table 1:** Children’s characteristics

	Overall (*n* = 841)	Boys (*n* = 424)	Girls (*n* = 417)	Boys vs. girls (mean difference or odds ratio (95% CI), p)
Demography				
Age (years)	10.2 (0.3)	10.2 (0.3)	10.2 (0.3)	0.0 (− 0.0–0.0), .803
Anthropometry				
Body mass (kg)	37.0 (8.1)	36.8 (7.8)	37.2 (8.3)	−0.26 (− 1.35–0.83), .641
Height (cm)	142.9 (6.7)	143.1 (6.7)	142.6 (6.8)	0.60 (−0.31–1.51), .197
BMI (kg/m^2^)	18.0 (3.0)	17.9 (2.9)	18.1 (3.1)	−0.25 (− 0.65–0.15), .218
Overweight and obese (%)	20.8	20.0	21.5	0.92 (0.68–1.25), .583*
Waist circumference (cm)	61.9 (7.5)	62.2 (7.3)	61.6 (7.7)	0.71 (−0.30–1.72), .169
Waist:height (ratio)	0.43 (0.05)	0.43 (0.05)	0.43 (0.05)	0.00 (−0.00–0.01), .322
Indices of cardiometabolic health				
Andersen test (m)	898 (103)	925 (112)	871 (85)	54 (41–68), < .001
Systolic blood pressure (mmHg)	105.2 (8.4)	105.3 (8.2)	105.2 (8.6)	0.29 (−0.82–1.40), .612
Diastolic blood pressure (mmHg)	57.7 (6.2)	57.4 (6.0)	58.1 (6.3)	−0.56 (− 1.38–0.26), .180
Total cholesterol (mmol/l)	4.46 (0.69)	4.46 (0.70)	4.46 (0.68)	0.00 (−0.09–0.09), .976
LDL-cholesterol (mmol/l)	2.51 (0.64)	2.50 (0.65)	2.53 (0.62)	−0.03 (− 0.11–0.06), .570
HDL-cholesterol (mmol/l)	1.59 (0.35)	1.63 (0.34)	1.55 (0.35)	0.08 (0.03–0.13), .001
Total:HDL-cholesterol (ratio)	2.91 (0.71)	2.82 (0.66)	2.99 (0.74)	−0.17 (−0.26–-0.07), .001
Triglyceride (mmol/l)	0.78 (0.38)	0.72 (0.31)	0.84 (0.42)	−0.13 (− 0.18–-0.08), < .001
Glucose (mmol/l)	4.98 (0.32)	5.02 (0.31)	4.94 (0.33)	0.08 (0.03–0.12), .001
Insulin (pmol/l)	7.91 (4.29)	7.05 (3.48)	8.33 (4.83)	−1.70 (−2.25–-1.15), < .001
HOMA (index)	1.71 (0.98)	1.54 (0.83)	1.89 (1.09)	−0.35 (−0.48–-0.22), < .001
Composite score (1SD)**	0.00 (1.00)	0.00 (0.93)	0.00 (1.07)	–
Physical activity (vertical axis)				
Wear time (min/day)	795 (56)	799 (59)	791 (54)	8.3 (0.7–15.9), .032
Overall physical activity (cpm)	708 (272)	754 (296)	660 (235)	82 (48–116), < .001
SED (min/day)	597 (56)	593 (59)	601 (53)	−13.2 (−17.9–-8.5), < .001
LPA (min/day)	122 (22)	124 (23)	120 (21)	2.5 (−0.2–5.2), .065
MPA (min/day)	37 (10)	39 (10)	35 (8)	4.1 (2.9–5.2), < .001
VPA (min/day)	39 (15)	43 (16)	35 (12)	6.6 (4.8–8.5), < .001
MVPA (min/day)	76 (23)	82 (24)	70 (19)	10.7 (7.9–13.4), < .001
Guideline amount (%)***	74	80	68	1.83 (1.40–2.40), < .001*

*BMI* body mass index, *LDL* low density lipoprotein, *HDL* high density lipoprotein, *HOMA* homeostasis model assessment, *SED* sedentary time, *LPA* light physical activity, *MPA* moderate physical activity, *VPA* vigorous physical activity, *MVPA* moderate-to-vigorous physical activity. *Odds ratio; **The composite score includes waist:height ratio, systolic blood pressure, total: HDL ratio, triglycerides, HOMA, and the Andersen test. Intensity-specific PA is calculated using the Evenson cut points [29]; ***The guideline PA levels is defined as a mean of ≥60 min of MVPA per day

### Correlation structure among physical activity intensity variables (explanatory variables)

Correlations among PA variables are shown for raw and clr-transformed variables in Additional file 1: Table S1 and Additional file 2: Table S2 (“traditional” description: four PA variables) and Additional file 3: Table S3 and Additional file 4: Table S4 (“spectrum” description: 23 PA variables). In the raw dataset, time spent SED (i.e., in the 0–99 cpm intensity interval) correlated moderately and negatively with all other variables, whereas all other variables were positively related to each other. The compositional datasets, however, showed very different correlation structures. Using the traditional description, SED and LPA were positively correlated to each other, but negatively related to MPA and VPA. Using the spectrum description, intensities from 0 to 4000 cpm (i.e., SED to MPA) were positively correlated to each other, but strongly negatively related to time spent ≥ 4000 cpm (VPA). Furthermore, correlations in compositional data weakened more rapidly from proximal to more distal variables than for raw data (e.g., r between 7000 and 7499 and 5000–5499 cpm = 0.21 versus 0.76 for compositional and raw data, respectively) (Additional file 3: Table S3 and Additional file 4: Table S4).

### Association patterns with cardiometabolic health

#### Traditional description

**Fig. 1:**
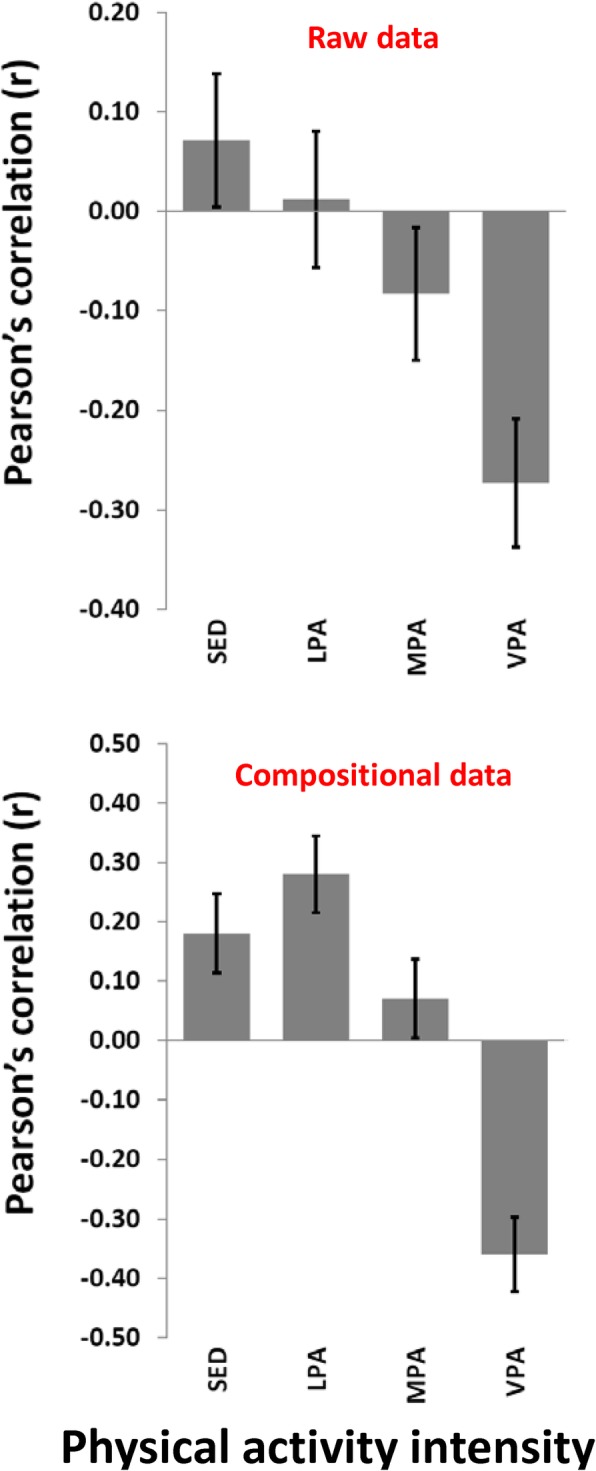
Bivariate correlations (95% confidence intervals) between physical activity intensities and a composite cardiometabolic health score using the traditional description of four physical activity variables. Raw data (upper panel), compositional data (lower panel)

**Fig. 2:**
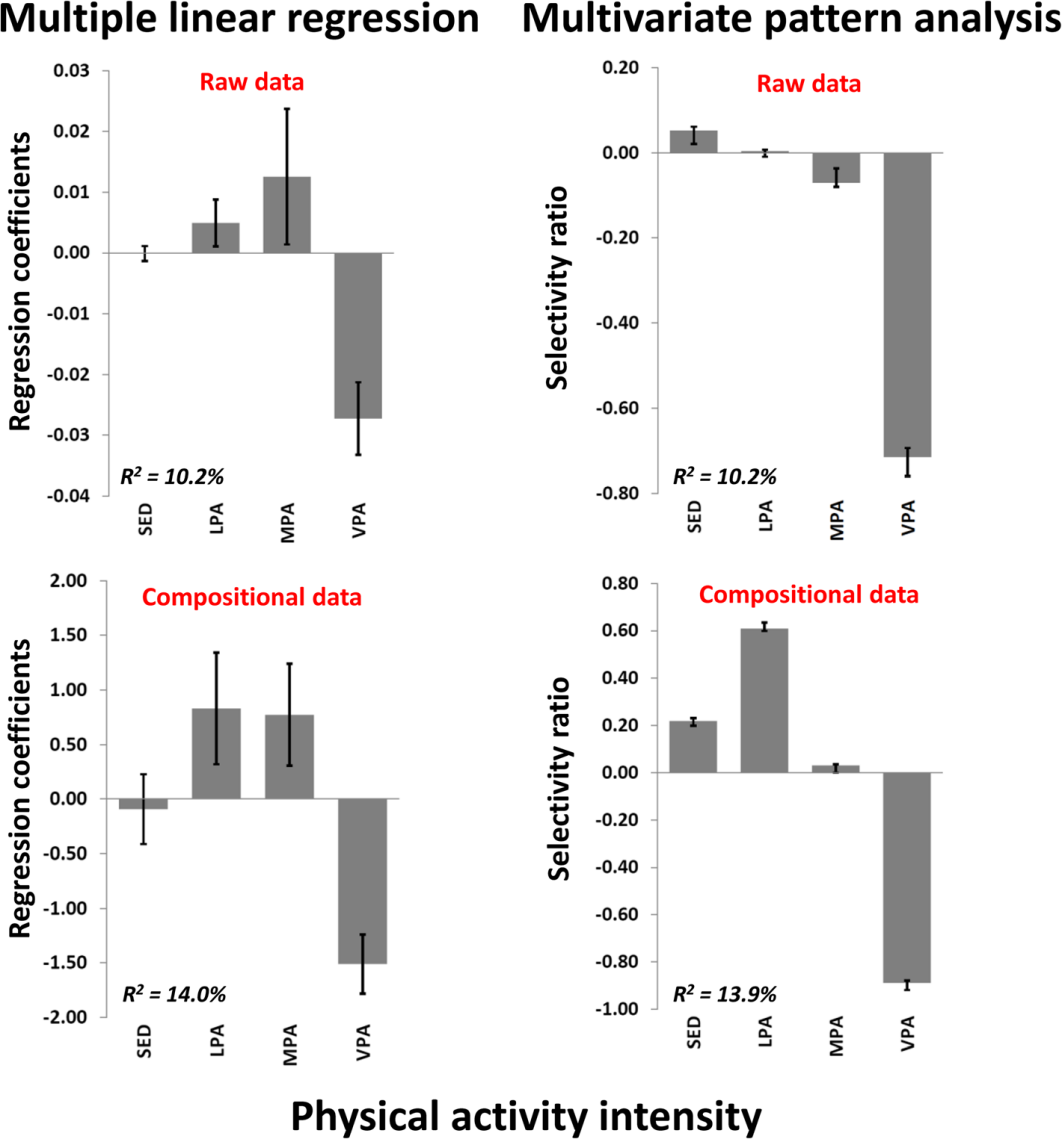
Association patterns between physical activity intensities and a composite cardiometabolic health score using the traditional description of four physical activity variables using different analytic approaches. Multiple linear regression with raw data (upper left panel), multiple linear regression with compositional data using the ilr-transformation (lower left panel), multivariate pattern analysis with raw data (upper right panel), and multivariate pattern analysis with compositional data using the clr-transformation (lower right panel). Selectivity ratio is calculated as the ratio of explained to total variance on the predictive (target projected) component. R^2^ = explained variance of the model

Figure 1 shows the bivariate correlation pattern between the traditional PA intensity variables (not mutually adjusted for each other) and the cardiometabolic health composite score. Note that a negative score implies better cardiometabolic health. Whereas weak positive associations were observed for SED and stronger negative associations were observed for VPA for both the raw and the compositional data, associations for LPA (no association versus positive association, respectively) and MPA (negative association versus positive association, respectively) differed. Figure 2 shows the associations between PA and cardiometabolic health using the four different analytic approaches. Explained variances ranged from 10.2 to 14.0% across the models. While VPA had a strong negative association with cardiometabolic health in all models, there were clear differences in the patterns of associations for other intensities between the models. Multiple linear regression of raw data and ilr-transformed data showed similar results, indicating statistically significant positive associations for both LPA and MPA with cardiometabolic health, and no associations for SED. Multivariate pattern analysis of both raw data and clr-transformed data, however, showed positive associations for SED. However, while a positive association was found for LPA and no association were found for MPA in the clr-transformed dataset, no association was found for LPA and a negative association was found for MPA in the raw dataset.

Association patterns with cardiometabolic health were similar using minutes/day and proportions of valid wear time in both the bivariate analysis and the multivariate pattern analysis (*r* > 0.99).

#### Intensity spectrum description

**Fig. 3:**
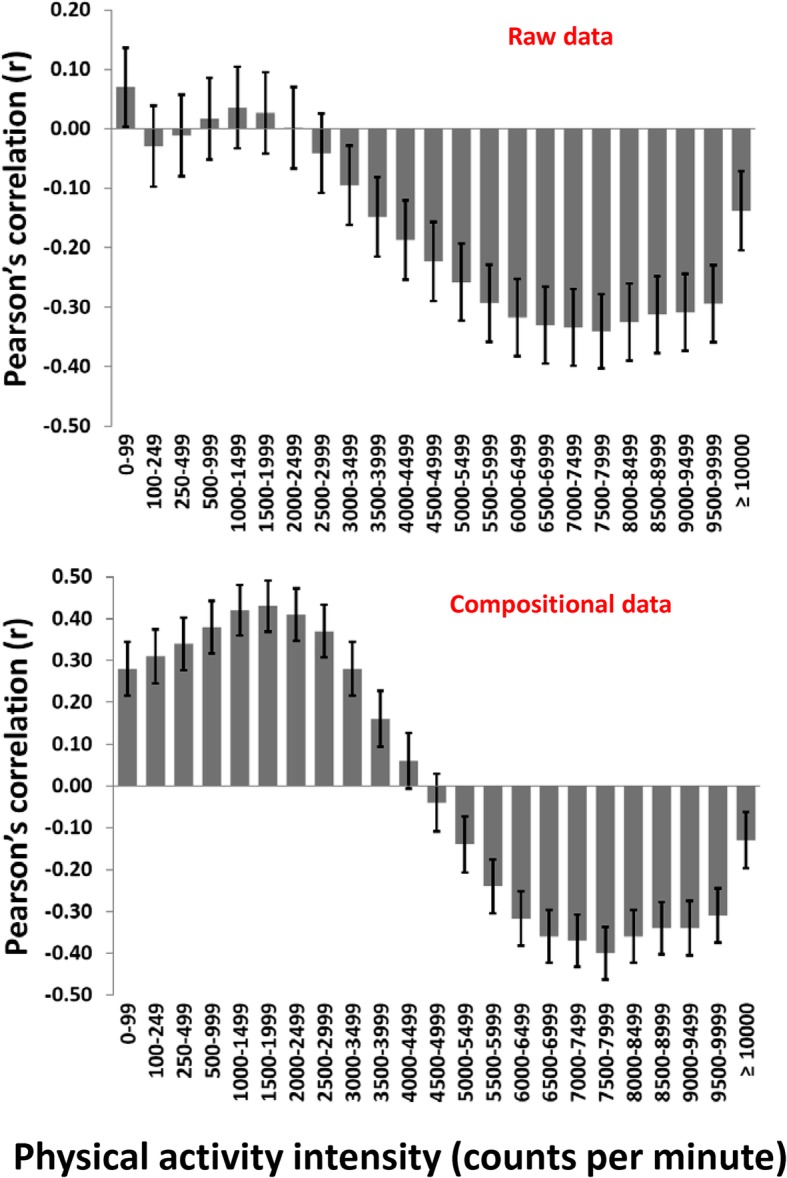
Bivariate correlations (95% confidence intervals) between physical activity intensities and a composite cardiometabolic health score using the spectrum description of 23 physical activity variables. Raw data (upper panel), compositional data (lower panel)

**Fig. 4:**
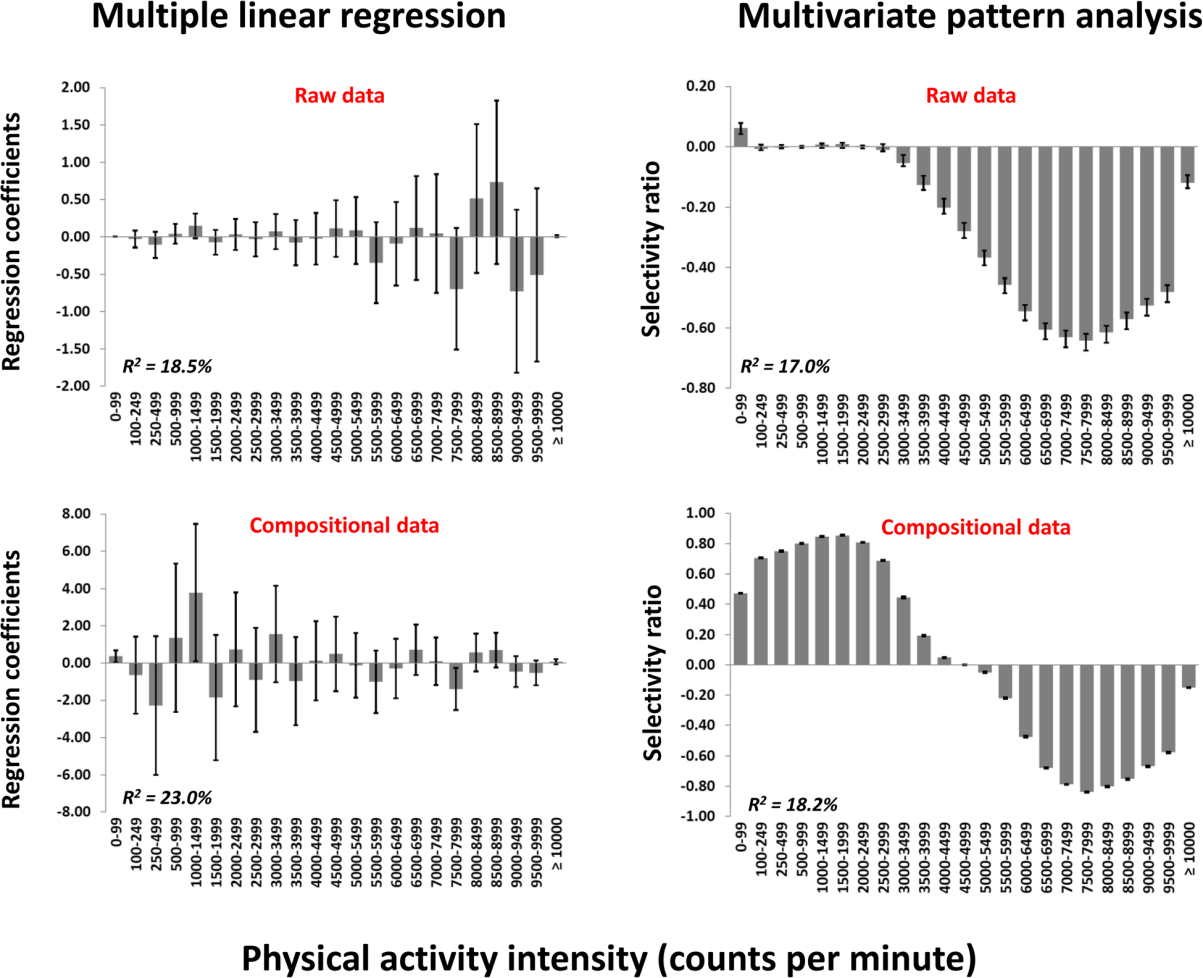
Association patterns between physical activity intensities and a composite cardiometabolic health score using the spectrum description of 23 physical activity variables using different analytic approaches. Multiple linear regression with raw data (upper left panel), multiple linear regression with compositional data using the ilr-transformation (lower left panel), multivariate pattern analysis with raw data (upper right panel), and multivariate pattern analysis with compositional data using the clr-transformation (lower right panel). Selectivity ratio is calculated as the ratio of explained to total variance on the predictive (target projected) component. R^2^ = explained variance of the model

Figure 3 shows the bivariate correlation pattern between the PA intensity spectrum variables (not mutually adjusted for each other) and cardiometabolic health. For raw data, a weak positive association was seen for 0–99 cpm, no associations were seen for intensities from 100 to 2999 cpm, whereas negative associations were seen for intensities ≥3000 cpm. For compositional data, positive associations were seen for intensities < 4000 cpm, whereas negative associations were seen for intensities ≥ 5000 cpm. The strongest negative associations were seen for intensities from 7000 to 7999 cpm. Figure 4 shows the association patterns between PA and cardiometabolic health using the four different analytic approaches. Explained variances ranged from 17.0 to 23.0% across the models. Both multiple linear regression models (for raw data and the ilr-transformed data) showed instable association patterns, as indicated by the fluctuating regression coefficients and large CIs. In contrast, multivariate pattern analysis provided stable association patterns for both the raw data and the clr-transformed data. Association patterns were, however, fundamentally different between the datasets. The association pattern for the raw data indicated a positive association for 0–99 cpm and gradually stronger negative associations for intensities ≥3000 cpm. For compositional data, however, all variables ≤4499 cpm were positively associated and all associations for variables ≥5000 cpm were negatively associated with cardiometabolic health.

Association patterns with cardiometabolic health were similar using minutes/day and proportions of valid wear time in both the bivariate analysis and the multivariate pattern analysis (*r* > 0.99).

## Discussion

Most studies using accelerometry-derived PA to examine associations with health-related outcomes include only a limited number of explanatory variables (e.g., SED, MVPA) to circumvent key issues regarding multicollinearity. However, this practice substantially reduces information and increases susceptibility to residual confounding [4–6]. Alternative methods have been proposed to address multicollinearity; however, these methods have key limitations that may result in conflicting conclusions. In the present analyses, we showed that different analytic approaches may lead to different association patterns of PA related to cardiometabolic health and thus conflicting conclusions regarding the importance of various PA intensities for cardiometabolic health.

Differences in associations between the analytical approaches were clear in both the traditional (SED, LPA, MPA, and VPA) and spectrum data descriptions (23 variables describing PA in much greater detail; 0–99 to ≥ 10,000 cpm). While VPA was consistently negatively (favorably) associated with the cardiometabolic health measure using the traditional description, both raw data and ilr-transformed data resulted in positive (unfavorable) associations for LPA and MPA with the cardiometabolic health score using multiple linear regression. In contrast, LPA was not related and MPA was negatively related to this score using bivariate correlations and the multivariate pattern analysis with raw data. Thus, multicollinearity is apparently a problem already with few variables. When having a greater number of and more strongly related variables using the larger spectrum description, these problems were further exaggerated. The unstable association patterns and the large CIs for both the raw data and the ilr-transformed data using this description, clearly suggest that linear regression is unsuitable for determining associations for multicollinear explanatory variables. This finding was expected, given that multiple linear regression cannot handle singularity or near singularity in the explanatory data matrix [8]. Although compositional data analysis seeks to solve the feature of reallocation of time among PA intensities resulting from closure, it does not solve the broader issue of multicollinearity that goes far beyond the impact of closure. Thus, our findings indicate that using ilr-transformation followed by multiple linear regression analysis [12, 13, 16] may lead to erroneous interpretation of associations between PA and cardiometabolic health. While this conclusion is less obvious for the traditional description of data, it is convincingly shown when multiple regression is applied to the spectrum description, for which the results are not interpretable.

Interestingly, explained variances were higher for compositional data than for raw data and higher for multiple regression than for multivariate pattern analysis models, particularly when using the spectrum description. We regard the effect of the compositional transformation on explained variance mainly a chance finding, caused by the alteration of the explanatory data structure (i.e., the associations could be stronger, similar, or lower, depending on the changes of the explanatory variables). Additionally, the higher explained variance of the linear regression model compared to the multivariate pattern analysis model could be a result of overfitting. While the multivariate pattern analysis uses cross-validation to estimate the number of components to be included in the models, linear regression do not include this procedure and might therefore be over fitted by including correlated noise that result in higher explained variance. Importantly, the cross-validation of the multivariate pattern analysis leaves out noise/irrelevant information from the explanatory variables, while this information is incorporated in the linear regression model, which means that the linear regression model includes noise correlated with the outcome variable. Although this difference partly could account for the discrepant results between the statistical approaches, we regard the great difference in handling the multicollinearity between the explanatory variables a much more influential difference: While linear regression seeks to delineate the unique variation with the outcome for each variable (i.e., establish independent associations), multivariate pattern analysis use latent variable modelling to exploit the variables’ correlated nature. Since the explanatory variables are strongly correlated, and therefore do not contribute uniquely to explain the outcome, the latter approach is arguably more meaningful, particularly when using the more informative spectrum description.

Most studies merge all intensities above MPA as MVPA [4], which gives the same weight to brisk walking and fast running and disregarding valuable information across the PA spectrum. Likewise, there may be important differences in associations with cardiometabolic health at the lower end of the PA spectrum [4, 39], which would be of public health importance. Thus, we and others contend that associations with cardiometabolic health for the whole PA intensity spectrum should be addressed to obtain a complete picture of these associations and facilitate more meaningful and valuable conclusions [4–7]. However, due to the strong multicollinearity between variables, novel statistical methods are needed to overcome this challenge [7, 9]. Aadland et al. [6, 14] have previously addressed the multicollinearity challenge of accelerometry-derived PA data using multivariate pattern analysis, which can treat accelerometry-derived PA variables as an intensity spectrum without respect to the number and distributions of variables being analyzed and the correlations among them without any transformation of data [20, 22, 23]. Thus, in contrast to previous studies that have applied compositional transformation using the ilr-method, which through a sophisticated set of transformations circumvent the closure problem and thus, in principle, allow for analysis by multiple linear regression [16], we were able to analyze singular compositional data using the clr-transformation and compare it with raw data. In contrast to the association pattern for raw data using multivariate pattern analysis, the compositional transformation substantially altered the association pattern with cardiometabolic health, as both SED, LPA and MPA using the ilr-transformation and the traditional description, and also MPA using the clr-transformation and the spectrum description (intensities up to 4500 cpm), were positively associated with poorer cardiometabolic health (i.e., a higher composite score). Thus, the association pattern revealed for compositional data contrasts previous studies and guidelines recommending intensities in the moderate range for improved health [1, 3, 4, 6, 14, 40]. In terms of informing guidelines and interventions, linear regression of both raw data and compositional (ilr-transformed) data suggest that MPA is detrimental to cardiometabolic health and should be reduced, while multivariate pattern analysis suggest that MPA is favorable to cardiometabolic health and should be promoted. While these associations were weak and possibly of minor importance using the traditional description, at least compared to the stronger and consistent negative associations for VPA, findings observed using the spectrum description (revealed from the multivariate pattern analysis for which the results were interpretable) clearly suggest that both LPA and MPA is detrimental to cardiometabolic health. These conflicting findings would therefore confuse the development of children’s guidelines for PA and hinder efforts to promote healthy activity behaviors during childhood.

The differential association pattern with cardiometabolic health between compositional and raw data is probably the result of an altered and distorted correlation structure among the explanatory PA variables induced by the compositional transformation [17, 18]. Although a similar picture is observed using the traditional and the spectrum descriptions, the impact of the transformation is clearer when using a larger number of accelerometry variables. For the raw data, time spent SED (i.e., in the 0–99 cpm intensity interval) correlated moderately negatively with all other variables, whereas all other variables were positively related to each other. Conversely, after the clr-transformation, SED, LPA, and MPA (i.e., intensities from 0 to 4000 cpm) correlated positively to each other, but strongly negatively to VPA (≥ 4000 cpm). Furthermore, when compared to the raw data, correlations weakened more rapidly from proximal to more distal variables. This finding is consistent with previous findings showing that log-transformation may induce non-linearity among the explanatory variables [19], and possibly with the outcome. Thus, consistent with previous studies [17, 18], we found substantial distortions of the correlation structures as a result of compositional transformation as applied to a PA dataset where means and variances differ greatly among variables. However, Skala [17] showed that closure of the dataset using an increased number of variables (≥ seven) limited the distortion of the correlation structure, as the correlation caused by closure was distributed over many variables. On this basis, we could expect the distortion would be negligible using our spectrum description. However, we found a substantially altered correlation structure also with many variables, caused by the additional log-transformation and centering of data [19].

The limitation of multiple linear regression to model multicollinear data applies to open data (i.e., min/day of PA intensities) as well as closed data, including when analyzed as proportions (i.e., percent of valid wear time) or according to an isotemporal substitution paradigm [10]. Dumuid et al. [13] suggest that the current evidence-base of associations between PA and health are erroneous and should be interpreted with caution because studies have ignored the compositional nature of PA data. Our findings suggest otherwise. Indeed, while compositional transformations may solve the smaller problem of the closed nature of PA data after normalizing to wear time, it does not solve the larger problem of multicollinearity between PA variables irrespective of closure. Moreover, it introduces an even larger problem by distortion of the correlation structure among PA variables accompanying the log-centering transformation. We therefore argue that multivariate pattern analysis may be a more favorable future direction in the analysis of associations between accelerometer-derived PA and health outcomes. This recommendation is based on well-known features of the different models with regard to their ability to handle multicollinear explanatory variables, and the finding of more plausible association patterns with cardiometabolic health resulting from this model. A key strength of multivariate pattern analysis is the use of latent variable modelling and thus the ability to model simultaneously multiple highly correlated variables. Thus, it uses and treats all available information together, resulting in stronger and stable models of *association patterns* (as indicated by the smaller CIs) compared to models attempting to delineate each variable’s *unique relation* to the outcome. However, in contrast to the conclusion by Dumuid et al. [13], our findings do not imply that the current evidence base, as derived from multiple linear regression of raw data, is flawed. Actually, we show that the unadjusted (bivariate) association patterns with cardiometabolic health were fairly similar to the association patterns of the multivariate pattern analysis, and that results were similar when analyzed as minutes per day or proportions of wear time. Our findings therefore indicate that the second-best option for analyzing PA data is to apply raw data and bivariate correlations or simple regression analyses. Bivariate correlation analysis does not solve the multicollinearity challenge, but simply do not need to take it into account. Thus, using a sub-optimal model (bivariate correlation analysis) seems to be a better option than using an erroneous model (multiple linear regression).

### Strengths and limitations

The main strength of the present study is the direct comparison of several different approaches used to analyze associations between PA and health in the prevailing literature. The use of the same dataset with a large sample of children allowed for robust and stable comparisons across the statistical approaches. Furthermore, we created both a traditional description of four gross PA categories, which is most commonly applied in the literature, and a much more detailed description, having 23 narrow intensity intervals across the intensity spectrum. Thus, our approach is applicable to previous studies that have used the common PA description, but also shows how the different analytic approaches compare when extended to a more fine-grained description of PA. Indeed, the higher resolution of the intensity spectrum description served to amplify the problems of the traditional description, which revealed important differences and pitfalls of the analytic approaches.

The cross-sectional design limits our ability to draw causal conclusions. It should also be kept in mind that use of other cohorts, for example spanning other age groups, and the use of other outcomes, could lead to other findings due to different correlation structures among the explanatory PA variables and/or different association patterns between PA intensities and outcomes. Further studies are therefore warranted to explore these analytic issues and extend our findings. Finally, we do not know the true association pattern between PA intensities and cardiometabolic health. Thus, our conclusions of which statistical approach provide the best results are based on knowledge of the features and limitations of the different statistical approaches and also which results that seems plausible based on our current understanding of the health-enhancing effects of PA.

## Conclusion

We found a consistent negative (favorable) association between VPA and the cardiometabolic health measure across the analytic approaches using the traditional description of four PA intensity variables. Otherwise, results from the different analytic approaches with regard to revealing associations between PA and cardiometabolic health in children differed substantially. Multiple linear regression lead to instable and spurious associations because the PA variables violated the assumption of noncollinearity between the exploratory variables. The log-ratio transformation in compositional data analysis lead to distortion of the correlation structure among the PA variables and thus a distorted association pattern with cardiometabolic health. Multivariate pattern analysis appeared to handle the raw PA data correctly, leading to plausible interpretations of associations between PA and cardiometabolic health. We recommend future studies using accelerometry apply multivariate pattern analysis without any transformation of PA data to develop the field of PA epidemiology.

## Additional files

Additional file 1: **Table S1.** Correlation matrix among raw traditional physical activity intensity variables. (PDF 66 kb)

Additional file 2: **Table S2.** Correlation matrix among clr-transformed traditional physical activity intensity variables. (PDF 66 kb)

Additional file 3: **Table S3.** Correlation matrix among raw spectrum physical activity intensity variables. (PDF 122 kb)

Additional file 4: **Table S4.** Correlation matrix among clr-transformed spectrum physical activity intensity variables. (PDF 125 kb)

## Data Availability

The datasets used in the current study are available from the corresponding author on reasonable request.

## Abbreviations

CLR: Centered log-ratio
CPM: Counts per minute
HDL: High-density lipoprotein cholesterol
HOMA: Homeostasis model assessment
ILR: Isometric log-ratio
LDL: Low-density lipoprotein cholesterol
LPA: Light physical activity
MPA: Moderate physical activity
MVPA: Moderate-to-vigorous physical activity
PA: Physical activity
PLS: Partial least squares
SBP: Systolic blood pressure
SED: Sedentary time
SR: Selectivity ratio
TC: Total cholesterol
TG: Triglyceride
VPA: Vigorous physical activity
