# Supplementary material for: Multicollinear physical activity accelerometry data and associations to cardiometabolic health: challenges, pitfalls, and potential solutions
Source: Int J Behav Nutr Phys Act. 2019 Aug 27;16:74. doi: 10.1186/s12966-019-0836-z (PMC6712694; doi:10.1186/s12966-019-0836-z)
Supplement: Supplementary file 1 — Table S1. Correlation matrix among raw traditional physical activity intensity variables. (PDF 66 kb) [file 12966_2019_836_MOESM1_ESM.pdf]

Table S1. Correlation matrix among raw traditional physical activity intensity variables.

	LPA	MPA	VPA
**SED**	-0.23	-0.38	-0.36
**LPA**		0.66	0.44
**MPA**			0.69

SED = sedentary time; LPA = light physical activity; MPA = moderate physical activity; VPA = vigorous physical activity. Grey area denotes negative correlations
