# Supplementary material for: Multicollinear physical activity accelerometry data and associations to cardiometabolic health: challenges, pitfalls, and potential solutions
Source: Int J Behav Nutr Phys Act. 2019 Aug 27;16:74. doi: 10.1186/s12966-019-0836-z (PMC6712694; doi:10.1186/s12966-019-0836-z)
Supplement: Supplementary file 2 — Table S2. Correlation matrix among clr-transformed traditional physical activity intensity variables. (PDF 66 kb) [file 12966_2019_836_MOESM2_ESM.pdf]

Table S2. Correlation matrix among clr-transformed traditional physical activity intensity variables.

	LPA	MPA	VPA
**SED**	0.23	-0.56	-0.73
**LPA**		-0.15	-0.66
**MPA**			0.05

SED = sedentary time; LPA = light physical activity; MPA = moderate physical activity; VPA = vigorous physical activity. Grey area denotes negative correlations
