# Supplementary material for: Multicollinear physical activity accelerometry data and associations to cardiometabolic health: challenges, pitfalls, and potential solutions
Source: Int J Behav Nutr Phys Act. 2019 Aug 27;16:74. doi: 10.1186/s12966-019-0836-z (PMC6712694; doi:10.1186/s12966-019-0836-z)
Supplement: Supplementary file 3 — Table S3. Correlation matrix among raw spectrum physical activity intensity variables. (PDF 122 kb) [file 12966_2019_836_MOESM3_ESM.pdf]

Table S3. Correlation matrix among raw spectrum physical activity intensity variables.

**PA intensity (cpm)**		**2**	**3**	**4**	**5**	**6**	**7**	**8**	**9**	**10**	**11**	**12**	**13**	**14**	**15**	**16**	**17**	**18**	**19**	**20**	**21**	**22**	**23**
**1**	**0-99**	-.06	-.11	-.18	-.26	-.33	-.36	-.37	-.36	-.34	-.34	-.34	-.33	-.33	-.31	-.31	-.30	-.29	-.29	-.28	-.28	-.28	-.25
**2**	**100-249**		.98	.91	.80	.68	.57	.46	.37	.30	.27	.27	.27	.27	.26	.25	.25	.23	.22	.21	.22	.21	.17
**3**	**250-499**			.97	.88	.76	.64	.53	.44	.36	.32	.32	.32	.32	.31	.29	.29	.27	.26	.26	.26	.26	.19
**4**	**500-999**				.96	.87	.75	.63	.52	.43	.39	.38	.39	.38	.37	.35	.34	.33	.32	.31	.32	.32	.25
**5**	**1000-1499**					.96	.87	.74	.61	.51	.46	.45	.45	.44	.43	.41	.40	.37	.36	.36	.37	.37	.29
**6**	**1500-1999**						.96	.85	.70	.58	.51	.50	.50	.49	.47	.44	.43	.40	.39	.38	.39	.38	.32
**7**	**2000-2499**							.95	.83	.69	.61	.57	.55	.54	.52	.49	.46	.43	.41	.40	.40	.39	.32
**8**	**2500-2999**								.95	.84	.74	.68	.65	.62	.59	.55	.51	.48	.45	.43	.42	.40	.31
**9**	**3000-3499**									.96	.88	.81	.76	.71	.66	.61	.57	.53	.49	.46	.44	.42	.30
**10**	**3500-3999**										.97	.92	.86	.80	.74	.67	.62	.57	.53	.50	.48	.45	.30
**11**	**4000-4499**											.98	.93	.87	.80	.73	.67	.62	.58	.55	.52	.49	.30
**12**	**4500-4999**												.98	.93	.87	.80	.74	.68	.63	.60	.57	.54	.32
**13**	**5000-5499**													.98	.93	.87	.81	.76	.71	.67	.64	.61	.35
**14**	**5500-5999**														.98	.94	.89	.83	.78	.74	.71	.67	.39
**15**	**6000-6499**															.98	.95	.90	.85	.81	.77	.73	.41
**16**	**6500-6999**																.98	.95	.91	.87	.83	.79	.45
**17**	**7000-7499**																	.98	.95	.92	.88	.84	.48
**18**	**7500-7999**																		.98	.96	.93	.89	.52
**19**	**8000-8499**																			.98	.96	.94	.56
**20**	**8500-8999**																				.98	.96	.60
**21**	**9000-9499**																					.98	.64
**22**	**9500-9999**																						.68
**23**	**≥10000**																						

PA = physical activity. Grey area denotes negative correlations
