# Supplementary material for: Multicollinear physical activity accelerometry data and associations to cardiometabolic health: challenges, pitfalls, and potential solutions
Source: Int J Behav Nutr Phys Act. 2019 Aug 27;16:74. doi: 10.1186/s12966-019-0836-z (PMC6712694; doi:10.1186/s12966-019-0836-z)
Supplement: Supplementary file 4 — Table S4. Correlation matrix among clr-transformed spectrum physical activity intensity variables. (PDF 125 kb) [file 12966_2019_836_MOESM4_ESM.pdf]

Table S4. Correlation matrix among clr-transformed spectrum physical activity intensity variables.

**PA intensity (cpm)**		**2**	**3**	**4**	**5**	**6**	**7**	**8**	**9**	**10**	**11**	**12**	**13**	**14**	**15**	**16**	**17**	**18**	**19**	**20**	**21**	**22**	**23**
**1**	**0-99**	.77	.75	.71	.64	.56	.47	.38	.26	.13	-.00	-.13	-.26	-.38	-.47	-.53	-.55	-.57	-.55	-.55	-.55	-.53	-.44
**2**	**100-249**		.99	.95	.88	.77	.63	.46	.27	.08	-.08	-.21	-.35	-.49	-.59	-.66	-.68	-.69	-.67	-.67	-.64	-.60	-.40
**3**	**250-499**			.98	.92	.81	.67	.50	.29	.09	-.08	-.21	-.36	-.50	-.62	-.68	-.70	-.72	-.70	-.69	-.65	-.61	-.41
**4**	**500-999**				.97	.88	.74	.55	.33	.10	-.07	-.22	-.37	-.51	-.64	-.71	-.73	-.75	-.72	-.71	-.66	-.62	-.41
**5**	**1000-1499**					.96	.84	.65	.40	.14	-.05	-.20	-.36	-.51	-.65	-.73	-.76	-.78	-.75	-.73	-.68	-.63	-.39
**6**	**1500-1999**						.95	.78	.51	.22	.00	-.16	-.32	-.49	-.64	-.73	-.77	-.79	-.77	-.74	-.70	-.65	-.37
**7**	**2000-2499**							.92	.69	.39	.14	-.05	-.24	-.42	-.59	-.69	-.75	-.78	-.77	-.76	-.72	-.68	-.38
**8**	**2500-2999**								.90	.65	.40	.17	-.06	-.28	-.48	-.61	-.70	-.75	-.77	-.76	-.75	-.72	-.42
**9**	**3000-3499**									.90	.71	.48	.22	-.05	-.29	-.47	-.59	-.67	-.71	-.72	-.72	-.71	-.45
**10**	**3500-3999**										.93	.76	.52	.23	-.04	-.27	-.43	-.52	-.59	-.62	-.63	-.64	-.44
**11**	**4000-4499**											.92	.74	.48	.20	-.07	-.24	-.37	-.45	-.50	-.52	-.54	-.41
**12**	**4500-4999**												.91	.72	.44	.16	-.05	-.20	-.31	-.37	-.40	-.44	-.38
**13**	**5000-5499**													.90	.69	.43	.21	.03	-.10	-.18	-.24	-.29	-.32
**14**	**5500-5999**														.88	.69	.49	.31	.16	.06	-.02	-.09	-.25
**15**	**6000-6499**															.89	.76	.61	.46	.34	.23	.14	-.15
**16**	**6500-6999**																.91	.81	.69	.57	.46	.36	-.02
**17**	**7000-7499**																	.92	.84	.75	.64	.54	.09
**18**	**7500-7999**																		.93	.87	.80	.70	.22
**19**	**8000-8499**																			.93	.88	.81	.32
**20**	**8500-8999**																				.93	.88	.42
**21**	**9000-9499**																					.92	.51
**22**	**9500-9999**																						.59
**23**	**≥10000**																						

PA = physical activity. Grey area denotes negative correlations
